# Elevated hydrostatic pressure triggers release of OPA1 and cytochrome C, and induces apoptotic cell death in differentiated RGC-5 cells

**Published:** 2009-01-19

**Authors:** Won-Kyu Ju, Keun-Young Kim, James D. Lindsey, Mila Angert, Ankur Patel, Ray T. Scott, Quan Liu, Jonathan G. Crowston, Mark H. Ellisman, Guy A. Perkins, Robert N. Weinreb

**Affiliations:** 1 The Sophie and Arthur Brody Optic Nerve Laboratory, Hamilton Glaucoma Center, University of California, San Diego, La Jolla, CA; 2 National Center for Microscopy and Imaging Research, School of Medicine, University of California San Diego, La Jolla, CA; 3 Glaucoma Investigation and Research Unit, University of Melbourne, Center for Eye Research Australia, Melbourne, Australia

## Abstract

**Purpose:**

This study was conducted to determine whether elevated hydrostatic pressure alters mitochondrial structure, triggers release of the dynamin-related guanosine triphosphatase (GTPase) optic atrophy type 1 (OPA1) or cytochrome C from mitochondria, alters *OPA1* gene expression, and can directly induce apoptotic cell death in cultured retinal ganglion cell (RGC)-5 cells.

**Methods:**

Differentiated RGC-5 cells were exposed to 30 mmHg for three days in a pressurized incubator. As a control, differentiated RGC-5 cell cultures were incubated simultaneously in a conventional incubator. Live RGC-5 cells were then labeled with MitoTracker Red and mitochondrial morphology was assessed by fluorescence microscopy. Mitochondrial structural changes were also assessed by electron microscopy and three-dimenstional (3D) electron microscope tomography. *OPA1* mRNA was measured by Taqman quantitative PCR. The cellular distribution of OPA1 protein and cytochrome C was assessed by immunocytochemistry and western blot. Caspase-3 activation was examined by western blot. Apoptotic cell death was evaluated by the terminal deoxynucleotidyl transferase dUTP nick end labeling (TUNEL) method.

**Results:**

Mitochondrial fission, characterized by the conversion of tubular fused mitochondria into isolated small organelles, was triggered after three days exposure to elevated hydrostatic pressure. Electron microscopy confirmed the fission and noted no changes to mitochondrial architecture, nor outer membrane rupture. Electron microscope tomography showed that elevated pressure depleted mitochondrial cristae content by fourfold. Elevated hydrostatic pressure increased *OPA1* gene expression by 35±14% on day 2, but reduced expression by 36±4% on day 3. Total OPA1 protein content was not changed on day 2 or 3. However, pressure treatment induced release of OPA1 and cytochrome C from mitochondria to the cytoplasm. Elevated pressure also activated caspase-3 and induced apoptotic cell death.

**Conclusions:**

Elevated hydrostatic pressure triggered mitochondrial changes including mitochondrial fission and abnormal cristae depletion, alteration of *OPA1* gene expression, and release of OPA1 and cytochrome C into the cytoplasm before the onset of apoptotic cell death in differentiated RGC-5 cells. These results suggest that sustained moderate pressure elevation may directly damage RGC integrity by injuring mitochondria.

## Introduction

Elevated intraocular pressure (IOP) is an important risk factor for optic nerve damage in glaucoma [1]. However, the precise pathophysiological relationship among elevated IOP, glaucomatous optic nerve damage and retinal ganglion cell (RGC) death remains poorly understood. Because mitochondrial changes have been identified in association with neuronal death in other models of central nervous system disease [2,3], it is possible that glaucomatous optic neuropathy may also involve mitochondrial changes. Recently, a double polymorphism in the optic atrophy type 1 (O*PA1*) gene has been linked with normal tension glaucoma [4]. However, the significance of these findings is controversial [4-10].

In healthy cells, mitochondria are autonomous and morphologically dynamic organelles that structurally reflect a precise balance of ongoing fission and fusion within a cell [11-14]. This balance is regulated by a family of dynamin-related guanosine triphosphatase (GTPases) that exert opposing effects. OPA1, the human ortholog of Mgm1p/Msp1p, and the mitofusins are required for mitochondria fusion. A dynamin-related protein (Drp-1) regulates mitochondrial fission [11,13]. Recent studies indicate that down-regulation of *OPA1* causes mitochondrial fission, leading to cytochrome C release and apoptosis in HeLa cells [15-17], as well as induces aggregation of the mitochondrial network in purified RGCs [18]. OPA1 has recently been identified as a dynamin-related GTPase which is involved in various processes related to mitochondrial inner membrane structural dynamics [19,20]. Mutations in *OPA1* are linked with neurodegenerative disease in human and cause autosomal dominant optic atrophy, a common hereditary optic neuropathy [19,20]. OPA1 is expressed in the soma and axons of the RGCs as well as in retinal horizontal cells [21-24]. The specific functional role of OPA1 in these cells remains unknown.

Emerging evidence suggests that OPA1 release during mitochondrial fission participates in apoptotic cell death [17,25]. However, little is known about the relationship between *OPA1* expression and distribution, and apoptotic cell death of RGCs in glaucoma. We hypothesized that elevated pressure, a major risk factor for glaucoma, may directly trigger mitochondrial structural changes associated with release of OPA1 and cytochrome C into the cytoplasm and induce apoptotic cell death in RGCs.

To address the issue, we determined whether elevated hydrostatic pressure alters mitochondrial structure and *OPA1* gene expression, triggers release of OPA1 or cytochrome C from mitochondria, and can directly induce apoptotic cell death in cultured RGCs.

## Methods

### Chemicals

All chemicals were from Sigma (St. Louis, MO) under otherwise noted.

### *Culture of RGC-5 cell*s

The immortalized rat RGC line, RGC-5 (provided by Drs. Neeraj Agarwal and Raghu Krishnamoorthy, University of North Texas Health Science Center, Fort Worth, TX), was cultured in Dulbecco’s modified eagle’s medium (DMEM) containing 10% fetal calf serum (FCS), 100 U/ml penicillin and 100 μg/ml streptomycin (Invitrogen, La Jolla, CA) at 5% CO_2_ and 37 °C [26,27].

### Differentiation of RGC-5 cells

Differentiation of RGC-5 cells was performed as previously described [26-28]. Briefly, non-differentiated cells were first seeded in the 100 mm tissue culture dishes at a density of 5x10^4^. After 4 h, when the cells attached to the dish, the dishes were rinsed with serum-free medium three to five times. The dishes were then incubated with DMEM without FCS for 24 h under the conditions described above. The medium was then changed to DMEM containing 10% FCS and supplemented with succinyl concanavalin A (sConA; 50 μg/ml), a nontoxic derivative of the lectin concanavalin A [26,28]. Three days after treatment with sConA, the cells were exposed to elevated hydrostatic pressure for day 1, 2, or 3 without changing the culture medium.

### Pressure system

A pressurized incubator was designed to expose the cells to elevated hydrostatic pressure [27,29-32]. The plexiglas pressure chamber was connected via a low-pressure two-stage regulator (Gilmont Instruments, Barnant Company, Barrington, II) to a certified source of 5% CO_2_/95% air (Airgas Inc., San Diego, CA). This arrangement provided constant hydrostatic pressure within ±1 mmHg and ranging from 0 to 200 mmHg. Gas to the chamber was warmed and humidified by bubbling through two liters of water. Both the water flask and the pressure chamber were maintained at 37 °C by placing them inside an electronically controlled conventional incubator. Gas flow of 70 ml/min was monitored using ball-type flow gauge regulated with a needle valve in the outlet circuit. Pressure was monitored using diaphragm-driven dial pressure gauge plumbed into the inlet circuit adjacent to the pressure chamber inlet. This pressure gauge was readable through a double-paned window present in the door of the incubator chamber. The key strengths of our device are that gas flow and pressure can be easily and accurately regulated to ±1 mmHg using the flowmeter and the low-pressure two-stage regulator.

### Analysis of mitochondrial morphology

After application of elevated hydrostatic pressure, mitochondria in the RGC-5 cells were labeled by the addition of MitoTracker Red CMXRos to the cultures (100 nM final concentration; Molecular Probes, Eugene, OR) and maintaining for 20 min in a CO_2_ incubator [27]. MitoTracker Red CMXRos is concentrated in active mitochondria by a process that is dependent upon mitochondrial membrane potential, i.e., accumulation is inhibited by Actinomycin A but not by rotenone. Previous double labeling studies with the MitoTracker Red and antibodies to the mitochondrial protein cytochrome C oxidase showed that MitoTracker Red specifically labeled mitochondria [33]. The cultures were subsequently fixed with 0.5% glutaraldehyde (Ted Pella, Redding, CA) in Dulbecco’s phosphate-buffered saline (DPBS) for 30 min at 4 °C and counterstained with Hoechst 33342 (1 µg/ml, Molecular Probes) in DPBS. Mitochondrial morphology was observed by fluorescence microscopy.

### Western blot analysis

For OPA1 and caspase-3 analyses, RGC-5 cells were homogenized in a glass-Teflon Potter homogenizer in lysis buffer containing 20 mM Hepes, pH 7.5, 10 mM KCl, 1.5 mM MgCl_2_, 1 mM EDTA, 1 mM EGTA, 1 mM DTT, 0.5% CHAPS plus complete protease inhibitors (Roche Biochemicals, Indianapolis, IN). The supernatant was mixed with SDS–PAGE sample buffer and boiled for 10 min. Equivalent amounts of protein (10 µg) for each sample were loaded onto 4%–12% pre-cast polyacrylamide gradient gels (Invitrogen, Carlsbad, CA). The proteins were electrotransferred to a nitrocellulose membrane in Tris-Glycine-Methanol transfer buffer. The membrane was blocked for 1 h at room temperature in phosphate buffer saline (PBS) containing 5% nonfat dry milk and 0.05% Tween-20 and then incubated for 15 h at 4 °C with monoclonal mouse anti-OPA1 antibody (H-300, 1:1,000; BD Transduction Laboratories, San Diego, CA) and polyclonal rabbit anti-cleaved caspase-3 antibody (Cell Signaling, Beverly, MA). The membrane was then rinsed with 0.05% Tween-20 in PBS four times and incubated for 2 h at room temperature with peroxidase-conjugated goat anti-mouse IgG (1:2,000; Bio-Rad, Hercules, CA). The blot was developed using chemiluminescence detection (ECL Plus; GE Healthcare Bio-Sciences, Picataway, NJ; used to according to the manufacturer’s recommendations). Images were analyzed by using a digital fluorescence imager (Storm 860; GE Healthcare Bio-Sciences). Band densities on the western blots were analyzed using ImageQuant TL Analysis software (GE Healthcare Bio-Sciences).

To assess the subcellular distribution of OPA1 and cytochrome C, the cytosolic and mitochondrial fractions were isolated from cultured RGC-5 cells by differential centrifugation (Mitochondrial Isolation Kit; Pierce, Rockford, IL, used according to the manufacturer's Dounce homogenizer procedure). For western blot analysis, mitochondria were lysed with 2% CHAPS in PBS (DC protein assay; Bio-Rad). The cytosolic and mitochondrial fraction proteins were mixed with SDS–PAGE sample buffer and boiled for 10 min. Equivalent amounts of protein (10 µg) for each sample were loaded onto 10% pre-cast polyacrylamide gradient gels (Invitrogen). The proteins were electrotransferred to a nitrocellulose membrane in Tris-glycine-methanol transfer buffer. The membrane was blocked for 1 h at room temperature in PBS containing 5% nonfat dry milk and 0.05% Tween-20, and then incubated for 15 h at 4 °C with primary antibody: monoclonal mouse anti-OPA1 antibody (1:1,000; BD Transduction Laboratories), monoclonal mouse anti-cytchrome C antibody (clone 7H8.2C12, 1:2,000; BD Biosciences PharMingen, San Diego, CA), monoclonal mouse anti-Actin antibody (Ab-1, 1:3,000; Calbiochem, La Jolla, CA), or polyclonal rabbit anti- voltage-dependent anion channel (VDAC) antibody (Ab-5, 1:1,000; Calbiochem). The actin or VDAC antibodies were used to confirm similar cytosolic or mitochondrial protein loading in each lane of the western blots for OPA1, respectively. The membrane was then rinsed with 0.05% Tween-20 in PBS four times and incubated for 1 h at room temperature with peroxidase-conjugated goat anti-mouse IgG, goat anti-rabbit IgG antibody (1:2,000; Bio-Rad), or peroxidase-conjugated goat anti-mouse IgM (1:5,000; Calbiochem). The blot was developed using chemiluminescence detection as described above and band densities were normalized using actin as cytosolic fraction calibrator and VDAC as mitochondrial fraction calibrator with ImageQuant TL Analysis software (GE Healthcare Bio-Sciences).

### Taqman quantitative-polymerase chain reaction

Differentiated RGC-5 cells were stored in RNA-later (Ambion Inc., Austin, TX) at −20 °C. Total RNA was extracted with Trizol (Invitrogen), purified on RNeasy mini columns (Qiagen, Valencia, CA), and treated with RNase-free DNase I (Qiagen). The RNA purity was verified by confirming that the OD 260nm/280nm absorption ratio exceeded 1.9 and cDNA was synthesized using SuperScript II first-strand RT–PCR kit (Invitrogen). *OPA1* expression was measured by Taqman quantitative polymerase chain reaction (qPCR, MX3000P; Stratagene, La Jolla, CA) using 50 ng of cDNA and 2X Taqman universal PCR master mix (Applied Biosystems, Foster City, CA). Amplification conditions included 95 °C for 10 min, 95 °C for 30 s, and 60 °C for 1 min for 40 cycles. Glyceraldehyde 3-phosphate dehydrogenase (*GAPDH*) expression was used as a normalizer for each sample. Primers for *GAPDH* and *OPA1* and the Taqman probe for *GAPDH* were designed using Primer Express 2.0 software (Applied Biosystems), obtained from Biosearch Technologies (Novato, CA). The probe for *OPA1* was obtained from the Roche Universal Probe Library (Roche Diagnostics, Mannheim, Germany, Table 1), and the optimal concentrations for probe and primers were determined using heart tissue. Samples without cDNA (no-template control) showed no contamination of primers and probe. Standard curves were constructed using nine twofold dilutions (50 ng-0.195 ng) for both the target (*OPA1*) and the endogenous reference (*GAPDH*). The samples were run in triplicates for target gene and reference gene *GAPDH* as an internal control. Each experimental condition was repeated 3 times by 3 separate qPCR runs.

**Table 1:** Real time PCR primer and probe sequences for *OPA1* and *GAPDH*

**Gene**	**Type**	**Sequences (5′–3′)**
*OPA1* rat (NM_133585)	Forward	CAGCTGGCAGAAGATCTCAAG
	Reverse	CATGAGCAGGATTTTGACACC
	Probe	Universal Probe Library probe #2
		Cat. # 04684982001
*GAPDH* rat (X02231)	Forward	GAACATCATCCCTGCATCCA
	Reverse	CCAGTGAGCTTCCCGTTCA
	Probe	CTTGCCCACAGCCTTGGCAGC

The number at the threshold level of log-based fluorescnence (C_t_) was computed automatically by the MX3000P software (Stratagene) and compared across all conditions. Delta C_t_ (∆C_t_) was calculated by subtracting the Normalizer C_t_ (*GAPDH*) from Target C_t_ (*OPA1*). Delta-Delta C_t_ (∆∆C_t_) was calculated by subtracting the ∆C_t_ calibrator (non-pressurized control cells) from the ∆C_t_ Sample (pressurized cells) [34]. Fold changes of OPA1 gene expression level have been converted into percentages (Table 2).

**Table 2:** Raw C_t_ values and relative expression levels for *GAPDH* and *OPA1* in differentiated RGC-5 cells following elevated hydrostatic pressure.

**Group number**	**Time**	**Passage number**	**Conditions**	**C_t_ values *GAPDH***	**C_t_ values *OPA1***	**ΔC_t_**	**ΔΔC_t_**	**Relative expression**
1	Day 2	34	Control	17.06	28.81	11.75	−0.57	Upregulation by 47.6%
			30 mmHg	18.82	30	11.18		
	Day 3		Control	17.96	29.17	11.21	0.62	Down-regulation by 37.5%
			30 mmHg	17.85	29.68	11.83		
2	Day 2	28	Control	16.82	28.36	11.54	−0.48	Upregulation by 36.5%
			30 mmHg	16.82	27.88	11.06		
	Day 3		Control	16.94	27.87	10.93	0.68	Down-regulation by 37.7%
			30 mmHg	17.35	28.96	11.61		
3	Day 2	30	Control	16.98	28.34	11.36	−0.27	Upregulation by 20.6%
			30 mmHg	16.84	27.93	11.09		
	Day 3		Control	18.16	28.51	10.35	0.5	Down-regulation by 31.5%
			30 mmHg	18.51	29.36	10.85		

This table showed that expression for *GAPDH* and *OPA1* was not dependent with passage number and GAPDH did not change with elevated hydrostatic pressure. The negative ∆∆C_t_ represents upregulation of gene expression and the positive ∆∆C_t_ represents down-regulation of gene expression.

### Electron microscopy

Differentiated RGC-5 cells were grown on 35 mm glass bottom culture dishes (MatTek, Ashland, MA). After exposure to elevated hydrostatic pressure, cultures were fixed with a 37 °C solution of 2% paraformaldehyde, 2.5% glutaraldehyde (Ted Pella) in 0.1 M sodium cacodylate (pH 7.4), and transferred to room temperature for 5 min, and then incubated for an additional 30 min on ice. Fixed cultures were then rinsed 3 times for 3 min each with 0.1 M sodium cacodylate plus 3 mM calcium chloride (pH 7.4) on ice and then post-fixed with 1% osmium tetroxide, 0.8% potassium ferrocyanide, 3 mM calcium chloride in 0.1 M sodium cacodylate (pH 7.4) for 60 min and then washed 3 times for 3 min with ice-cold distilled water. Cultures were finally stained overnight with 2% uranyl acetate at 4 °C, dehydrated in graded ethanol baths, and embedded in Durcupan resin (Fluka, St. Louis, MO). Ultrathin (70 nm) sections were post-stained with uranyl acetate and lead salts and evaluated by a JEOL 1200FX transmission electron microscopy (EM) operated at 80 kV. Images were recorded on film at 8,000 magnification. The negatives were digitized at 1,800 dpi using a Nikon Cool scan system, giving an image size of 4033×6010 pixel array and a pixel resolution of 1.77 nm [27,33].

### Electron microscope tomography

Sections from in situ embedded monolayers of differentiated RGC-5 cells were cut at thicknesses of 400–500 nm. Sections were then stained 30 min in 2% aqueous uranyl acetate, followed by 15 min in lead salts. Fiducial cues consisting of 15 nm and colloidal gold particles were deposited on opposite sides of the section. For each reconstruction, a series of images at regular tilt increments was collected with a JEOL 4000EX intermediate-voltage electron microscope operated at 400 kV. The specimens were irradiated before initiating a tilt series to limit anisotropic specimen thinning during image collection. Tilt series were recorded on film at 20,000 magnification with an angular increment of 2° from −60° to +60° about an axis perpendicular to the optical axis of the microscope using a computer-controlled goniometer to increment accurately the angular steps. The illumination was held to near parallel beam conditions and optical density maintained constant by varying the exposure time. The negatives were digitized with a Nikon CoolScan (Nikon Inc., Melville, NY) at 1,800 dpi producing images of size 4033×6010 pixels. The pixel resolution was 0.7 nm. The IMOD package was used for rough alignment with the fine alignment and reconstruction performed using the TxBR package (NCMIR, UCSD, La Jolla, CA) [35]. Volume segmentation was performed by manual tracing in the planes of highest resolution with the program Xvoxtrace (NCMIR, UCSD) [36]. The mitochondrial reconstructions were visualized using Analyze (Mayo Foundation, Rochester, MN) or the surface-rendering graphics of Synu (NCMIR, UCSD) as described by Perkins et al. [36]. These programs allow one to step through slices of the reconstruction in any orientation and to track or model features of interest in three dimensions. Measurements of mitochondrial outer, inner boundary, and cristae membrane surface areas and volumes were made within segmented volumes by the programs Synuarea and Synuvolume respectively (NCMIR, UCSD). These were used to determine the cristae density, defined as the ratio:sum of the cristae volumes divided by the mitochondrial volume. Movies of the tomographic volume were made using Amira (Visage Imaging, Inc., Carlsbad, CA).

### Immunocytochemistry

For cytochrome C immunocytochemistry, RGC-5 cells were fixed with 0.5% glutaraldehyde (Ted Pella) for 30 min at 4 °C to preserve mitochondrial morphology [21]. Cells were then briefly rinsed with PBS and incubated with 1% sodium borohydride for 30 min at room temperature to quench auto-fluorescence. After permeabilization with 0.1% Triton X-100/PBS for 15 min at room temperature, cells were washed with PBS and then incubated with mouse anti-cytochrome C monoclonal antibody (clone 6H2.B4, 1:100; BD Biosciences PharMingen) for 16 h at 4 °C. After rinsing in PBS for 30 min, sections were incubated with a secondary antibody, goat-anti mouse IgG conjugated with Alexa Fluor 594 (1:200; Molecular Probes) for 4 h at 4 °C. After PBS wash, the sections were incubated with Hoechst 33342 (1 µg/ml; Molecular Probes) for 5 min at room temperature. For quantification of cytochrome C release, at least 250 cells were scored for each experimental condition. Results were expressed as a percentage of cells with completely released cytochrome C in the cytoplasm. For Thy1.1 and brain-derived neurotrophic factor (BDNF) immunocytochemistry, RGC-5 cells were fixed with 4% paraformaldehyde for 30 min at 4 °C. After permeabilization with 0.1% Triton X-100/PBS for 15 min at room temperature, cells were washed with PBS and then incubated with mouse anti-Thy1.1 monoclonal antibody (Clone OX-7, 1:500; Millipore, Billerica, MA) and rabbit anti- BDNF polyclonal antibody (1:500; Millipore) for 16 h at 4 °C. After rinsing in PBS for 30 min, sections were incubated with secondary antibodies, goat-anti mouse IgG or goat-anti rabbit IgG conjugated with Alexa Fluor 488 (1:200; Molecular Probes) for 4 h at 4 °C.

### TUNEL assessment of apoptosis

Following exposure to elevated hydrostatic pressure, RGC-5 cells were fixed with a freshly prepared 4% paraformaldehyde in PBS for 1 h at room temperature, rinsed with PBS, and then processed using a standard protocol for TUNEL staining (In Situ Cell Death Detection Kit; Roche Diagnostics, used according to the manufacturer's recommendations). The cells were then washed twice and were incubated with Hoechst 33342 (1 µg/ml; Molecular Probes) for 5 min at room temperature.

### Morphological assessment of apoptosis

Cells were grown on 12 mm coverslips in 24 well plates and exposed to elevated hydrostatic pressure for 3 days. Nuclei were stained with Hoechst 33342 (1μg/ml; Molecular Probes; 10 min at room temperature) and visualized using the fluorescence microscopy. Each evaluated cells was scored as having normal or pynotic (apoptotic) nuclei in several fields. At least 200 cells were scored for each experimental condition. Results were expressed as a percentage of cells with apoptotic nuclei among total cells counted.

Images were captured by fluorescence microscopy using a Nikon ECLIPSE microscope (E800; Nikon Instruments Inc., Melville, NY) equipped with digital camera (SPOT; Diagnostic Instrument, Sterling Heights, MI). Images were acquired using emission filters of 457 nm or 528 nm or 617 nm, collected by Simple PCI version 6.0 software (Compix Inc., Cranberry Township, PA), and exported as Photoshop files (Adobe Photoshop; Adobe, San Jose, CA).

### Statistical analysis

Experiments presented were repeated at least three times with triplicate samples. The data are presented as the mean±SD. Comparison of two experimental conditions was evaluated using the paired or unpaired Student’s *t*-test. A p<0.05 was considered to be statistically significant.

## Results

### Elevated hydrostatic pressure induces mitochondrial fission and leads to abnormal cristae depletion

**Figure 1:**
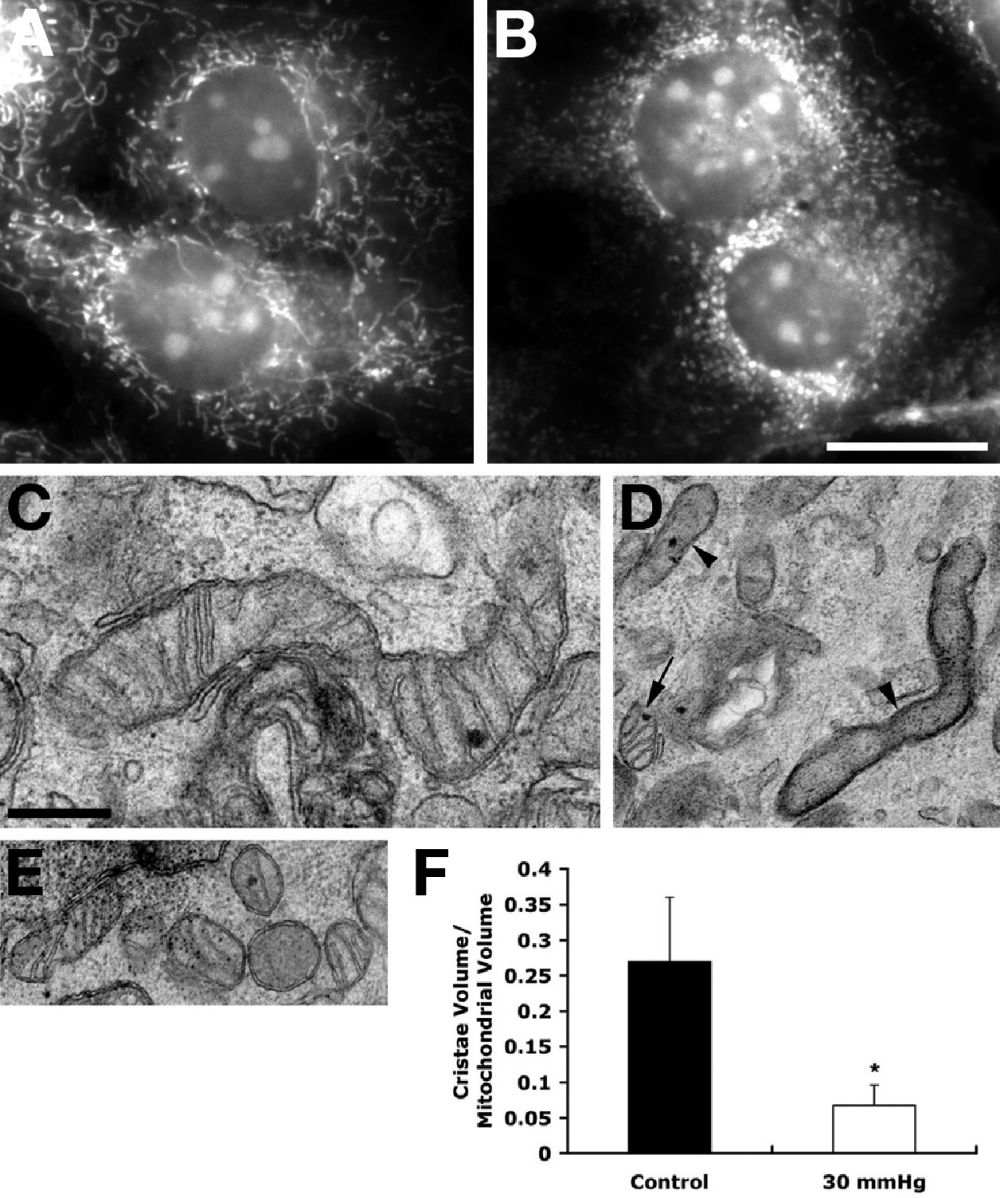
Mitochondrial fission and loss of cristae following exposure to elevated hydrostatic pressure. Differentiated RGC-5 cells were exposed to elevated hydrostatic pressure (30 mmHg) for 3 days and stained with MitoTracker Red. Non-pressurized control cells show a typical filamentous and fused mitochondrial network (**A**). Pressurized cells show mitochondrial fission, which is characterized by the conversion of tubular fused mitochondria into isolated small organelles (**B**). Electron micrographs of thin sections of RGC-5 cells show normal, elongated forms of mitochondria in non-pressurized control cells (**C**), whereas elevated hydrostatic pressure produces smaller mitochondria (arrow) and mitochondria with abnormal, severe cristae depletion (arrowheads; **D**). Closely apposed smaller mitochondria were also observed, such as the six shown in panel **E**, suggesting that fission had occurred. To quantify the observation of severe cristae depletion in the mitochondria exposed to elevated hydrostatic pressure, the cristae density in tomographic volumes was determined after segmentation. This parameter was calculated by dividing the sum of the cristae volumes by the mitochondrial volume for each mitochondrion (**F**). There were 13 control (from two experiments) and 14 pressurized mitochondria (from two experiments) fully segmented into constituent compartments and analyzed (The asterisk indicates significance at p<0.001 compared to non-pressurized cells). Data represent the means±SD. Size bar represents 20 µm (**A** and **B**) and 500 nm (**C-E**).

**Figure 2:**
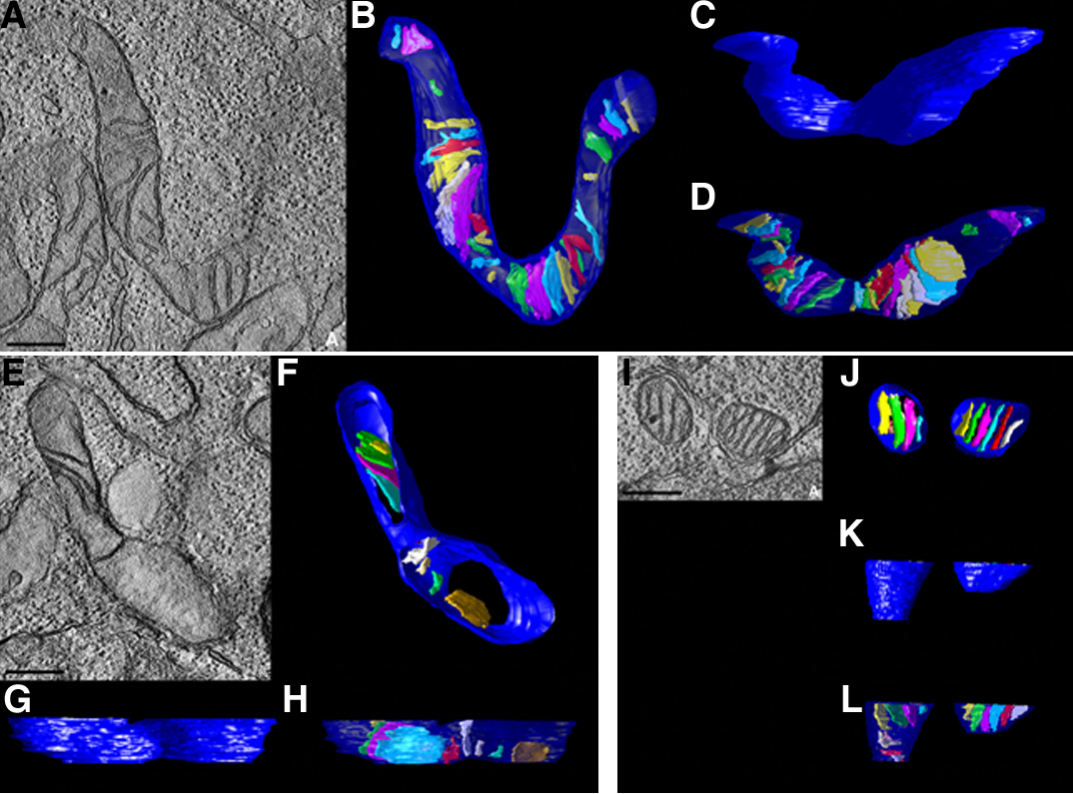
3D reconstructions of abnormal mitochondrial structure following exposure to elevated hydrostatic pressure. Differentiated RGC-5 cells were exposed to elevated hydrostatic pressure (30 mmHg) for 3 days. Non-pressurized cells show an elongated and fused mitochondrial network (**A-D**). Pressurized cells show abnormal cristae depletion (**E-H**), and mitochondrial fission (**I-L**). Mitochondria in these cells were reconstructed in 3D using electron microscope tomography. Slices of 1.4 nm thickness through the middle of each reconstruction are shown (**A**, **E**, **I**). Top and side views of surface-rendered volumes of a control mitochondrion and three pressurized mitochondria are displayed. The outer membrane is shown in blue and the cristae are shown in various colors. Top views of the mitochondria after segmentation of the outer and inner membranes into separate objects show the distribution of cristae (**B**, **F**, **J**). Side views of the outer membrane show no ruptures of the membrane (**C**, **G**, **K**). With the outer membrane made translucent (**D**, **H**, **L**), the cristae shapes and sizes are visualized. Also apparent is the extent to which the mitochondria volume is filled (or otherwise depleted) of cristae. Size bar represents 250 nm. Movie one (left) shows non-pressurized RGC-5 cells and movie 2 (right) shows pressurized RGC-5 cells. Double-click on the image to play the video. Note that the slide bar at the bottom of the quicktime movie can be used to manually control the flow of the movie. If you are unable to view the movie, a representative frame from the movie is included.

In agreement with our previous study [27], mitochondria in non-pressurized control cells at 3 days showed a typical filamentous and fused mitochondrial network (Figure 1A,C). In contrast, mitochondrial fission, characterized by the conversion of tubular fused mitochondria into isolated small round organelles, was induced at 3 days after elevated hydrostatic pressure (Figure 1B,D,E). Transmission EM analysis of images collectively showing several hundred mitochondria provided evidence that non-pressurized control cells contained classical long tubular mitochondria with abundant cristae (Figure 1C). In contrast, cells exposed to elevated hydrostatic pressure contained elongated mitochondria that had few cristae (Figure 1D) and a mix of small round mitochondria (Figure 2E). Again, EM images showing hundreds of mitochondria were analyzed. To gain insight into the mitochondrial ultrastructure of non-pressurized versus pressurized cells, cultures were fixed to preserve mitochondrial morphology, and processed by electron microscope (EM) tomography, to obtain 3D reconstruction showing detailed mitochondrial ultrastructure. Quantative analysis of tomographic volumes showed that pressure treatment induced a fourfold reduction of cristae density (Figure 1F). Tomographic reconstructions of control samples showed an intact outer mitochondrial membrane (OMM; blue) and lamellar cristae (various colors), occupying the mitochondrial matrix space (Figure 2B-D). The tomographic reconstruction of pressurized cells was dramatically different in two ways (Figure 2E-L). First, cristae depletion was often observed. Figure 2F-H shows a typical example of a mitochondrion that is devoid of cristae in much of its volume. Second, mitochondrial fission was apparent. Mitochondria of pressurized cells, were sometimes observed to be much smaller and more globular and in close proximity (Figure 2J-L), suggesting recent fission before fixation. The movies (**movie 1** and **movie 2**) have been made to illustrate the above reconstructions. These can be viewed in Figure 2.

### Elevated hydrostatic pressure alters *OPA1* mRNA level but did not affect OPA1 protein expression

**Figure 3:**
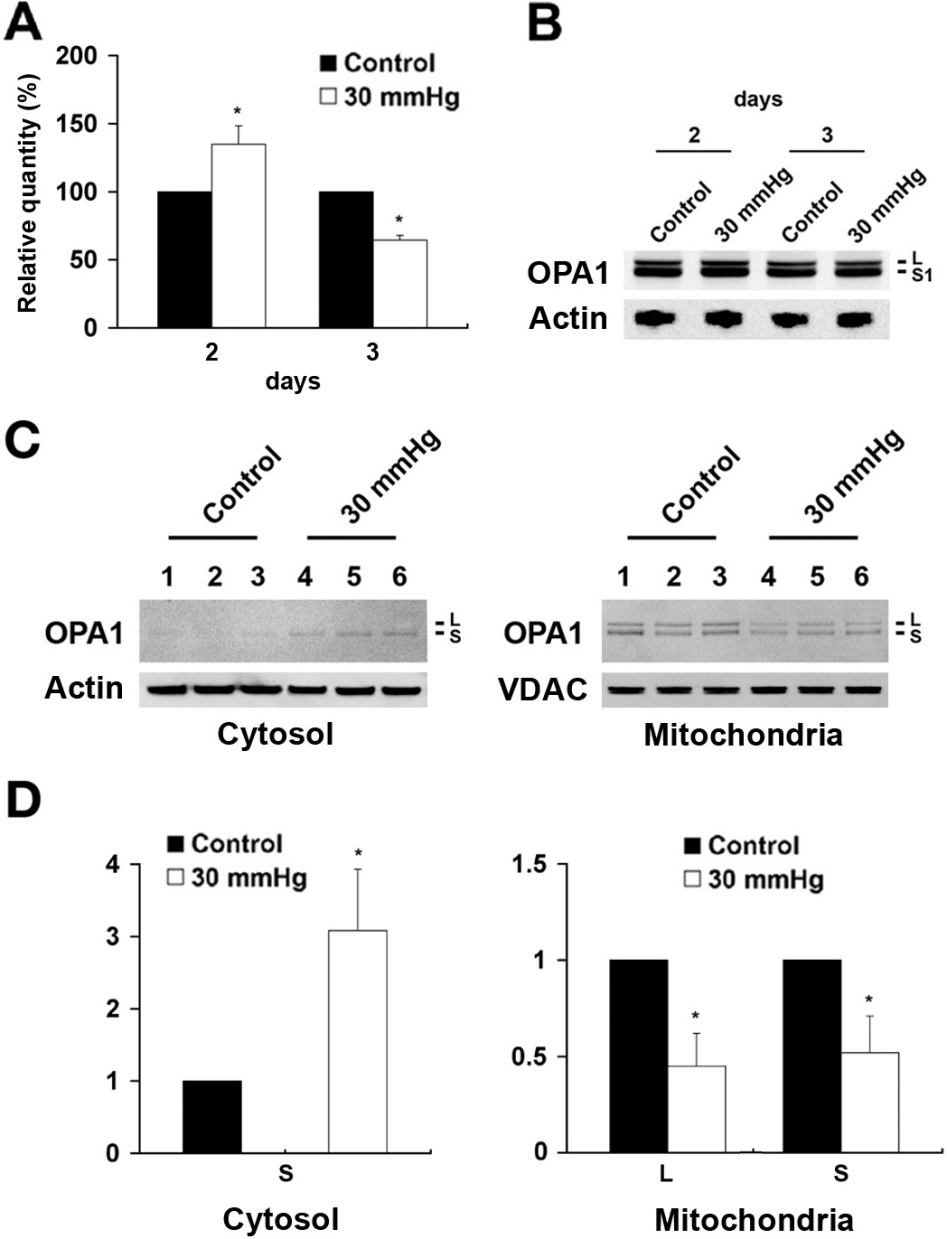
Alteration of *OPA1* gene and protein expression following exposure to elevated hydrostatic pressure. Following exposure to 30 mmHg for 2 and 3 days, the level of *OPA1* mRNA was measured with specific primers and probes for rat *OPA1* and *GAPDH* using Taqman qPCR and the level of total OPA1 protein was also measured using western blot. *OPA1* expression was significantly decreased in pressurized cells (**A**), compared control cells. However, OPA1 protein expression did not show a difference between control and pressure treated cells (**B**). Three protein samples from three independent experiments were loaded for each group. The OPA1 protein bands show the positions, based on comparison with size standards, of approximately the 80 (S) to 90 kDa (L) forms of OPA1. The blot was stripped and reprobed with anti-actin antibody (~42 kDa) to confirm similar protein loading in each lane (**B**). Mitochondria were separated from cytosol by differential centrifugation and OPA1 content was analyzed by western blotting. An 80 kDa isoform of OPA1 was observed in the cytosolic fraction whereas 80 and 90 kDa isoforms of OPA1 were observed in the mitochondrial fraction (**C**). Relative intensity of chemiluminescence for each protein band was normalized using actin as cytosolic fraction calibrator and VDAC as mitochondrial fraction calibrator. (**D**). The blot was stripped and reprobed with anti-actin antibody (~42 kDa) for cytosol fraction and anti-VDAC antibody (~31 kDa) for mitochondria fraction to confirm similar protein loading in each lane using same gel and exposure. Data represent the means±SD of three independent experiments.

To determine *OPA1* mRNA levels in differentiated RGC-5 cells exposed to elevated hydrostatic pressure and in non-pressurized control cells, specific primers and probes for rat *OPA1* and *GAPDH* were designed for Taqman qPCR (Table 1). As shown in Figure 3, the *OPA1* mRNA level was significantly increased by 34.9±13.5% at 2 days and reduced by 35.5±3.5% at 3 days after elevated hydrostatic pressure (n=3, Figure 3A and Table 2). We evaluated statistical significance in *GAPDH* mRNA level between control and experimental groups at days 2 and 3 after elevated hydrostatic pressure. Paired *t*-test showed that p values were 0.47 at day 2 and 0.32 at day 3, respectively, indicating that the differences were insignificant. Further, we also examined the pressure-induced changes in *OPA1* mRNA expression (∆C_t_) at days 2 and 3. Paired *t*-test analysis showed significantly increased *OPA1* gene expression at day 2 (p=0.0384) and significantly decreased at day 3 (p=0.0076). In western blots, the OPA1 antibody recognized two major bands in both non-pressurized control and pressurized RGC-5 cells with approximate molecular weights of 80 and 90 kDa (Figure 3B). These molecular weights agree with previously published data [21]. Interestingly, there was no significant difference in total OPA1 protein expression in differentiated RGC-5 cells exposed to elevated hydrostatic pressure for 2 or 3 days, compared to non-pressurized control cells, (Figure 3B). The blots were reprobed with actin antibody to confirm equal protein loading.

### Elevated hydrostatic pressure triggers OPA1 release from mitochondria

To assess whether elevated hydrostatic pressure triggers OPA1 release during mitochondrial fission, we examined subcellular localization of OPA1 in differentiated RGC-5 cells exposed to elevated hydrostatic pressure and in non-pressurized control cells. As shown in Figure 3, pressure treatment increased cytosolic OPA1 by 3.08±0.85 fold at 3 days. The single major band had an approximate molecular weight of 80 kDa (S) in the cytosolic fraction. In contrast, there were 80 kDa (S) and 90 kDa (L) OPA1 bands in the mitochondrial fraction. Following 3 days of elevated pressure, relative OPA1 content of two isoforms in the mitochondrial fraction were decreased by 0.45±0.17 fold (L) and 0.52±0.19 fold (S), respectively (n=3, Figure 3C,D). Both the 80 kDa and 90 kDa bands were reduced similarly in mitochondrial fraction. Because there was no change in the total OPA1 in the cells (Figure 3B), the increased cytoplasmic OPA1 and decreased mitochondrial OPA1 most likely reflect OPA1 release from the mitochondria. This is consistent with the reduced 80 kDa OPA1 in the mitochondrial fraction which was the same size as the cytoplasmic OPA1 (Figure 3C,D). In addition, degradation of 90 to the 80 kDa OPA1 form may have been induced because there was less of the 90 kDa OPA1 form, as well. Together these observations suggest that elevated hydrostatic pressure induced substantial OPA1 release from mitochondria to the cytosol. It is possible that the small release of OPA1 in the control cultures may reflect serum deprivation. However, this small release of OPA1 was not associated with mitochondrial fission in control cells until after 6 days of differentiation as observed previously [27]. The blots were reprobed with actin or VDAC (~31 kDa) antibody to confirm equal protein loading. VDAC is a well known constituent of the outer membrane of mitochondria [17,37-39].

### Elevated hydrostatic pressure causes cytochrome C release from mitochondria

**Figure 4:**
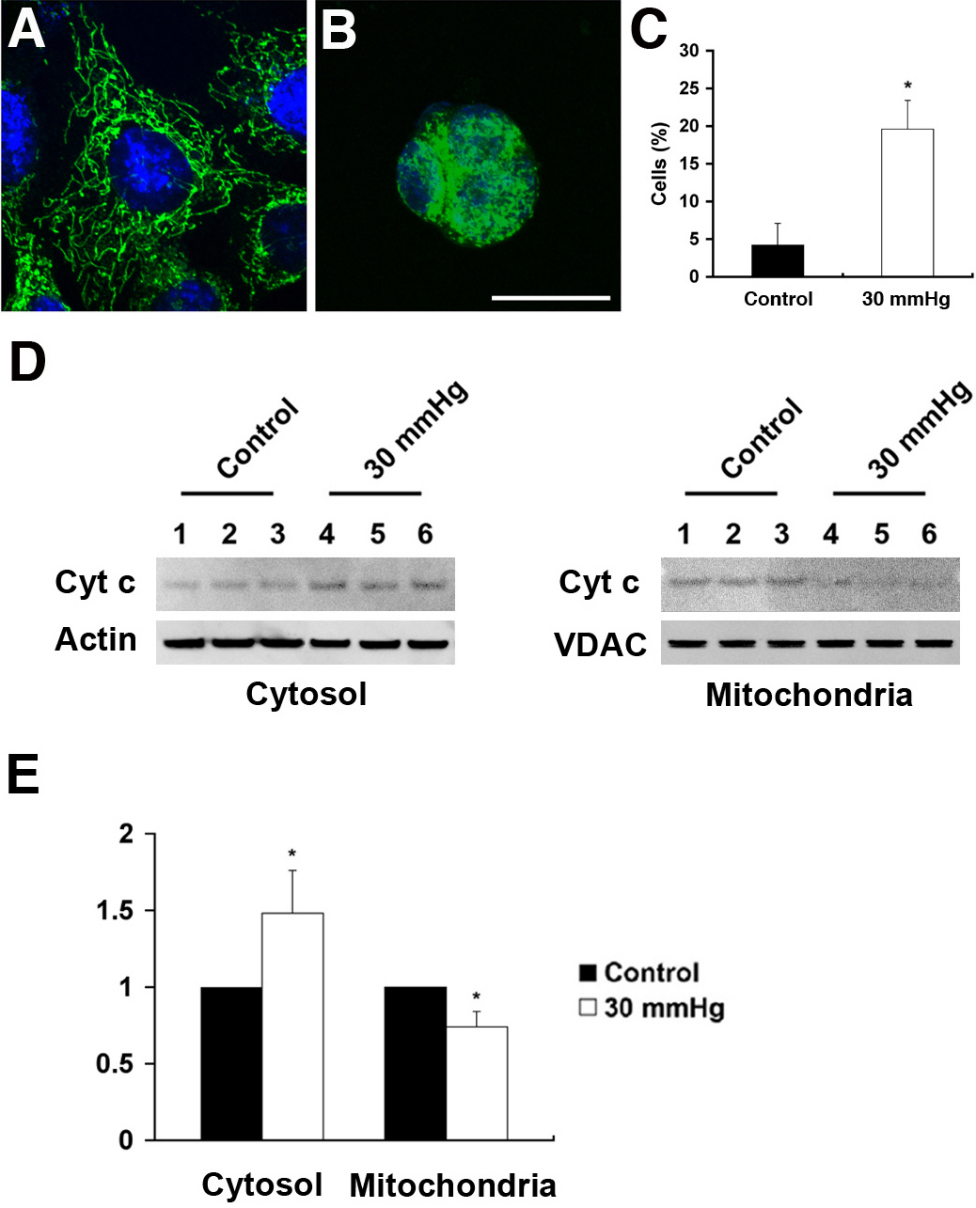
Cytochrome C release following exposure to elevated hydrostatic pressure. Differentiated RGC-5 cells were exposed to elevated hydrostatic pressure (30 mmHg) for 3 days and immunostained with cytochrome c antibody. Non-pressurized control cells show a typical filamentous and fused mitochondrial network (**A**). However, pressure treatment resulted in dispersed cytochrome C immunoreactivity in the cytoplasm (**B**). Size bar represents 10 µm (**A** and **B**). To quantify the observation of cytochrome C release from mitochondria exposed to elevated hydrostatic pressure, the percentage of cells with completely released cytochrome C in the cytoplasm was determined (**C**). Mitochondria were separated from cytosol by differential centrifugation and cytochrome C content was analyzed. Three protein samples from three independent experiments were loaded for each group. The cytochrome C protein bands show the positions, based on comparison with size standards, of the 15 kDa form of cytochrome C in cytosolic and mitochondrial fraction. The blot was stripped and reprobed with anti-actin antibody (~42 kDa) for cytosol fraction and anti-VDAC antibody (~31 kDa) for mitochondria fraction to confirm similar protein loading in each lane (**D**). Relative intensity of chemiluminescence for each protein band was normalized using actin as cytosolic fraction calibrator and VDAC as mitochondrial fraction calibrator (**E**). Data represent the means±SD of three independent experiments.

Cytochome C immunoreactivity was prominent within the mitochondria of non-pressurized control RGC-5 cells (Figure 4A). Pressure treatment for 3 days resulted in dispersed cytochrome C immunoreactivity throughout the cytoplasm (Figure 4B). To assess cytochrome C release after elevated hydrostatic pressure, we counted cells with completely released cytochrome C in the cytoplasm. The percentage of control cells with completely released cytochrome C in the cytoplasm was 4.2±2.9% and the percentage of pressure-treated cells with completely released cytochrome C in the cytoplasm was 19.6±3.8% at 3 days (n=3, Figure 4C). Parallel experiments analyzed using western blotting found that pressure treatment increased cytochrome C release by 1.48±0.28 fold, compared to the non-pressurized control cells. In contrast, cytochrome C content in the mitochondrial fraction was decreased by 0.74±0.10 fold after 3 day of elevated hydrostatic pressure (n=3, Figure 4D,E).

### Elevated hydrostatic pressure induces apoptotic cell death

**Figure 5:**
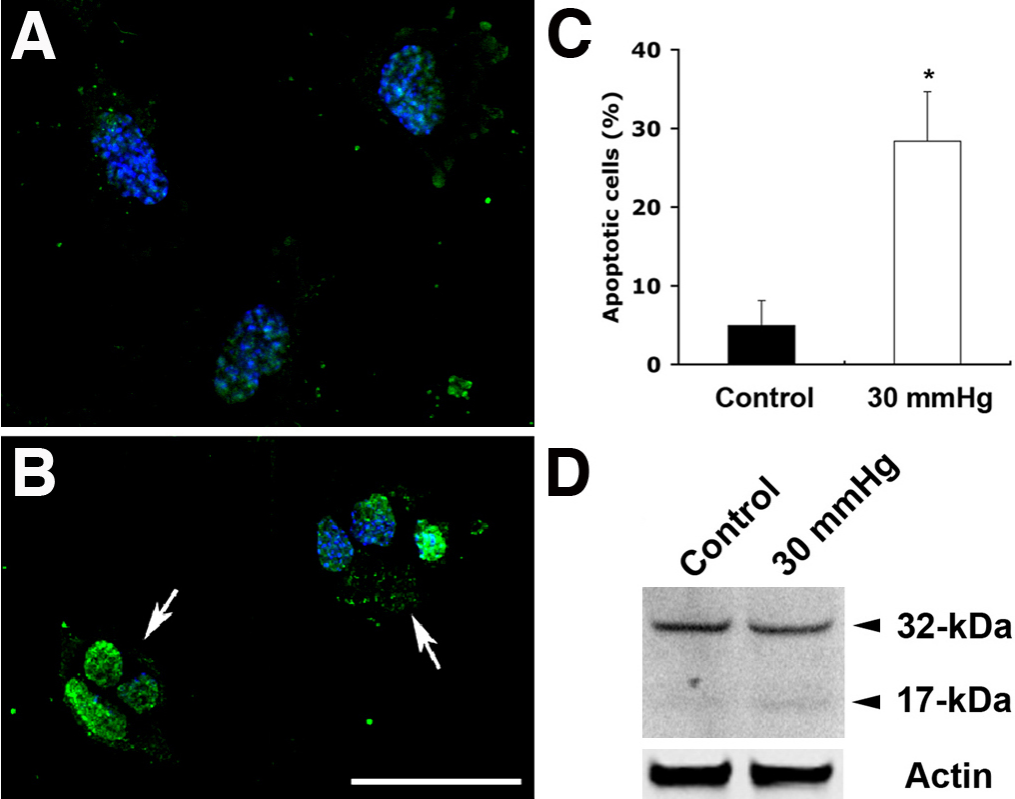
Apoptotic cell death following exposure to elevated hydrostatic pressure. Differentiated RGC-5 cells were exposed to elevated hydrostatic pressure (30 mmHg) for 3 days. Non-pressurized control cells show normal nuclei (**A**). However, the apoptotic phenotype of nuclei in RGC-5 cells exposed to elevated hydrostatic pressure was observed (arrows; **B**). Size bar represents 20 µm (**A** and **B**). Normal and apoptotic nuclei of RGC-5 cells were stained with Hoechst 33342 and counted in several fields under the fluorescence microscope (for ultraviolet excitation). Fifteen thousand cells in control and pressurized cells were counted, respectively, and shown as a percentage of cells with apoptotic nuclei among the total cells counted. Data represent the means±SD of three independent experiments (**C**). Caspase-3 activation was analyzed by western blotting. The presence of caspase-3 activation was assessed by the observation of 17 kDa subunit that derived from the cleavage of the 32 kDa pro-enzyme caspase-3 (**D**). The blot was stripped and reprobed with anti-actin antibody (~42 kDa) to confirm similar protein loading in each lane.

**Figure 6:**
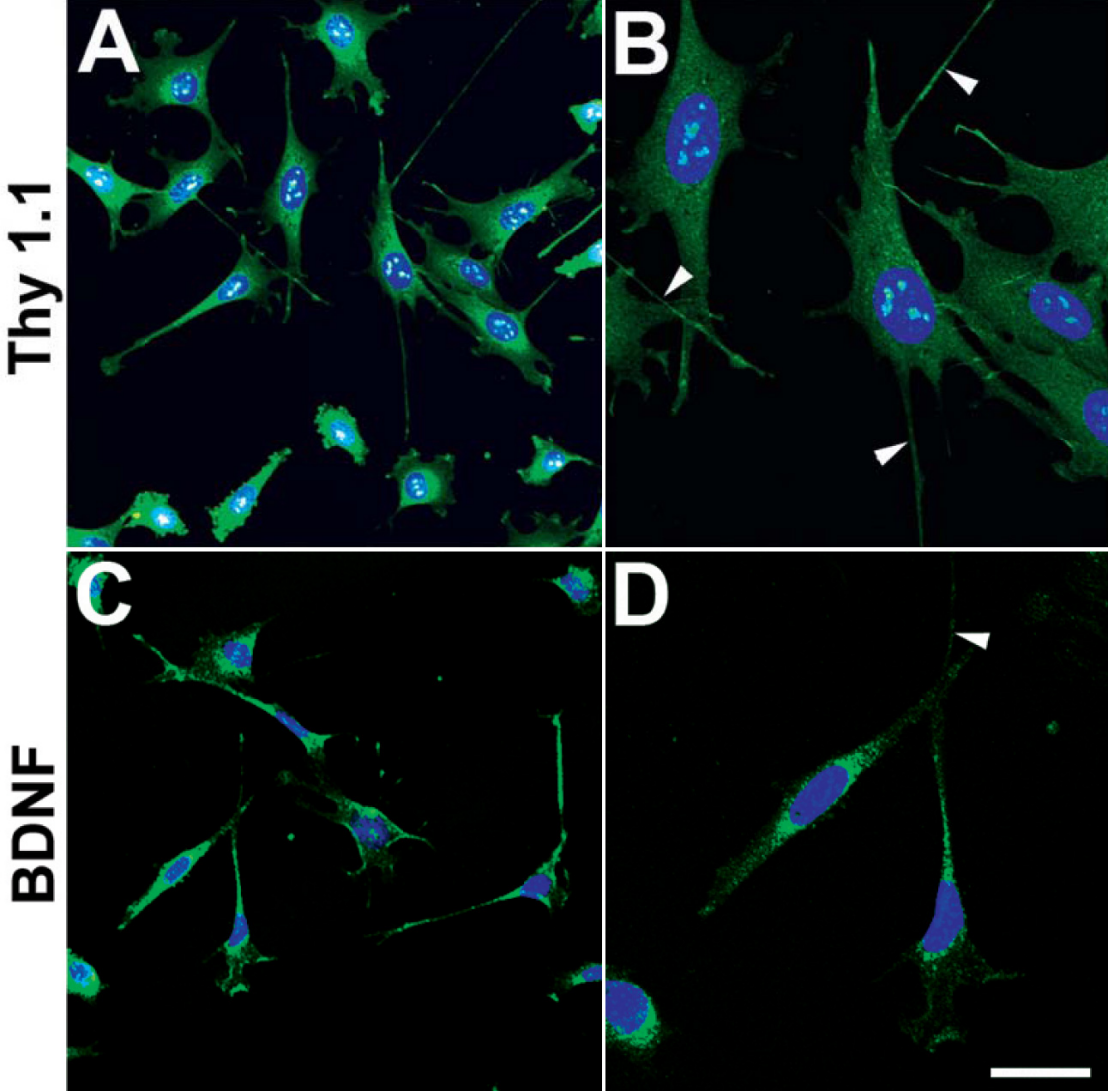
Thy1.1 and BDNF immunocytochemistry in differentiated RGC-5 cells. Thy1.1 (**A**) and BDNF (**C**) immunoreactivity are present in differentiated RGC-5 cells. Higher magnification showed that Thy1.1 (**B**) and BDNF (**D**)-positive RGC-5 cells extend neurites (arrowheads). Size bar represents 20 µm (**B** and **D**).

Apoptosis was induced in differentiated RGC-5 cells exposed to elevated hydrostatic pressure for 3 days. Specific morphological changes of apoptotic cell death included cell body shrinkage and compaction of the nucleus with Hoechst, compared to non-pressurized control cells (Figure 5). The TUNEL-positive apoptotic cells showed bright green labeling of fragmented nuclear DNA (Figure 5B), compared to non-pressurized control cells. To assess apoptotic cell death after elevated hydrostatic pressure, we counted apoptotic-condensed nuclei with Hoechst. The percentage of cells with condensed nuclei appearance was approximately 28.4±6.2% in the cultures exposed to elevated hydrostatic pressure at 3 days, compared to 4.9±3.1% in the non-pressurized control cells (n=3, Figure 5C). Using western blot, RGC-5 cells exposed to elevated hydrostatic pressure showed cleavage of caspade-3 at 3 days. The presence of caspase-3 activation was assessed by the observation of 17 kDa subunit that derived from the cleavage of the 32 kDa proenzyme caspase-3. In contrast, no cleavage of caspase-3 was detected in non-pressurized control cells (Figure 5D). Additionally, we confirmed that differentiated RGC-5 cells expressed BDNF and Thyl.1 as well as extended neurites (Figure 6).

## Discussion

These data demonstrate that elevated hydrostatic pressure induced substantial changes in mitochondrial fission, abnormal cristae depletion, altered *OPA1* expression, and induced release of both OPA1 and cytochrome C. These events may precede apoptotic cell death. Together, these results suggest that elevated hydrostatic pressure can induce alteration of *OPA1* gene expression and protein distribution, and apoptotic cell death in RGC degeneration. Although our model suggests the notion that mitochondrial fission following elevated hydrostatic pressure is linked to OPA1 release and apoptotic cell death in cultured RGC-5 cells, direct evidence of signal mechanisms between mitochondrial fission and OPA1 release following increased pressure still is lacking. Further, the OPA1 release-mediated upstream signaling mechanisms of apoptotic cell death following increased pressure also need to be investigated.

In the present study, differentiated RGC-5 cells expressed BDNF and Thyl.1 as well as extended neurites (Figure 6). The RGC-5 cell line used for this investigation is a transformed RGC line that has certain characteristics of RGCs including expression of Thy-1, BDNF, Brn-3C, neuritin, NMDA receptor, GABA-B receptor, and synaptophysin [26]. These cells do not express glial fibrillary acidic protein, HPC-1, or 8A-1; markers for astrocytes, amacrine cells, or horizontal cells and ganglion cells, respectively.

Previously, we observed that 3 days after exposure to elevated hydrostatic pressure produced insignificant alterations of pH, pCO_2_ or pO_2_ (1.01%, 1.03%, and 2.5%, respectively, p>0.5). Hence, it is unlikely that pressure-induced changes in pH, pCO_2_ or pO_2_ explain the dramatic mitochondrial changes we observed. Our findings agree with studies using similar pressure chamber designs with other cell types showing a negligible impact on these parameters within the culture media [29-31]. Several recent investigations reported that elevated hydrostatic pressure induces apoptotic death in RGC-5 cells as well as in primary cultures of purified RGCs [27,32,40-43]. It is not clear whether elevated hydrostatic pressure is specific to RGC-5 cells because elevated hydrostatic pressure also can induce apoptotic cell death in other cell lines such as B35 and PC12 [31]. Conversely, Tezel, and Wax [43] observed that elevated hydrostatic pressure induced apoptosis in RGCs but not in glial cells after the incubation of the co-cultures of RGCs with glial cells. These findings make it difficult to conclude that elevated pressure is specific for RGC-5 cells. However, it is likely that elevated pressure increases the susceptibility to damage of RGCs and make them more likely to die following axon damage at the optic nerve head.

Emerging evidence indicates that mitochondrial morphology and dynamics play an important role in cell and animal physiology. An imbalance in the control of mitochondrial fusion and fission dramatically alters overall mitochondrial morphology [11,13]. Recent evidence suggest that the mitochondrial fission machinery actively participates in the process of apoptosis and that excessive mitochondrial fission leads to breakdown of the mitochondrial network, loss of mitochondrial DNA, respiratory defects and an increase in reactive oxygen species in mammalian cells [11,15,16,39,44,45]. In addition, it has been reported that inhibiting Drp-1, which regulates mitochondrial fission, before inducing apoptosis not only inhibits mitochondrial fission but also delays caspase activation and the process of cell death itself [13,39,46-48]. Although the precise molecular pathways mediating mitochondrial fission in apoptosis remain unknown, it is possible that mitochondrial fission itself may be the effect as well as the cause of cellular stress.

Previously, we have been reported that elevated hydrostatic pressure caused breakdown of the mitochondrial network by mitochondrial fission [27]. Briefly, mitochondrial fission, characterized by the conversion of tubular fused mitochondria into isolated small organelles, was triggered in more than 74.3±1.9% of mitochondria at 3 days after elevated hydrostatic pressure. Only 4.7±1.4% of non-pressurized control cells displayed mitochondrial fission after 3 days [27]. Further, we have been also reported that elevated hydrostatic pressure induced abnormal cristae depletion and decreased length of mitochondria [27]. This suggests that mitochondria in which cristae are depleted in response to pressure may have bioenergetic impairment [27,33,49-51]. In the present study, we additionally found that the mitochondria of non-pressurized control cells had more than fourfold the number of cristae than mitochondria of pressurized cells. Thus, we propose that pressure treatment can induce changes of shape and length of mitochondrial cristae during mitochondrial fission as an upstream signaling event that leads to RGC degeneration.

Recent studies have been demonstrated that OPA1 protein was expressed in RGCs of the mouse, rat and human retinas [21,23,24] and that down-regulation of *OPA1* causes aggregation of the mitochondrial network in purified RGCs [18]. The present study demonstrated that *OPA1* mRNA and protein were expressed in differentiated RGC-5 cells. Interestingly, we observed that the expression level of *OPA1* mRNA was significantly increased before the onset of excessive mitochondrial fission but significantly decreased during mitochondrial fission following elevated hydrostatic pressure. This result is a good agreement with previous studies that down-regulation of OPA1 gene is linked to mitochondrial fission, mitochondrial cristae depletion and bioenergetic impairment [11,15-18,25]. It has been reported that many stimuli can modulate mRNA stability, most prominently, heat shock, ultraviolet (UV) radiation, hypoxia, and nutrient deprivation [52-58]. Because pressure treatment significantly alters *OPA1* mRNA level, it is possible that elevated pressure may be one of the candidates to modulate the stability of *OPA1* mRNA in differentiated RGC-5 cells. Alternatively, it is also possible that the turnover of OPA1 protein expression may be slower than alteration of *OPA1* mRNA biosynthesis. Further insight into the mechanism of these signaling cascades may be gained by directly studying *OPA1* mRNA stability or biosynthesis.

In the present study, we found that pressure treatment induces OPA1 release from mitochondria to the cytoplasm in differentiated RGC-5 cells. It has been reported that the release of only a small pool of OPA1 corresponding to a fraction of protein is present in the intermembrane space (IMS) of untreated mitochondria [25]. Gottlieb et al. has been demonstrated that a rhomboid intramembrane protease, presenilin-associated rhomboid-like (PARL), cleaves the OPA1 protein and that the cleavage of OPA1 generates a pool of truncated OPA1 that is soluble in the IMS [59]. Moreover, it has been suggested that soluble OPA1 is crucial for the anti-apoptotic effects of PARL because it maintains the bottleneck configuration of cristae and the comparmententalization of cytochrome C [59,60]. Recently, it has been reported that OPA1 consists of at least five isoforms in other types of cells including HeLa cells [60-63]. In the present study, we also found that an ~80 kDa OPA1 immunoreactive band mainly appeared in the cytosolic fraction in differentiated RGC-5 cells following elevated hydrostatic pressure. Significantly, we found that OPA1 has at least four isoforms in the cytosolic fraction of the rat retinas after acute IOP elevation using the same gel system in a separate study [64]. Thus, the detection of only two isoforms of OPA1 in differentiated RGC-5 cells may be because they are the predominant (if not the only) isoforms in this system. It is likely that the ~80 kDa OPA1 may be one of the truncated forms of OPA1 that may be soluble in the IMS. However, the possible functional role of the soluble OPA1 that is released from mitochondria in differentiated RGC-5 cells following elevated hydrostatic pressure needs to be further explored. In addition, detailed investigation of potential proteolytic processing of OPA1 should be addressed in future experiments.

It also has been reported that loss of OPA1 from mitochondria perturbates mitochondrial inner membrane structure and integrity, leading to cytochrome C release and apoptosis [15,25]. Further, this is a growing body of evidence, suggesting that release of OPA1 during apoptosis is associated with the rapid and complete release of cytochrome C and subsequent mitochondrial fragmentation [17,25]. These results are consistent with our findings that elevated hydrostatic pressure triggers release of both OPA1 and cytochrome C during apoptotic cell death. Furthermore, recent evidence has also shown that Drp-1 is essential for cytochrome C release [60]. Consistent with this evidence, we have previously reported that pressure treatment triggers Drp-1 translocation into the mitochondria in differentiated RGC-5 cells following elevated hydrostatic pressure [27]. Thus, we propose that pressure treatment may induce cytochrome C release by either unknown OPA1 or Drp-1-dependent mechanisms in our model. Several key experiments have shown that cytochrome C release can lead to caspase activation [65-67]. In the present study, cytochrome C release on day 3 of pressure treatment was correlated with caspase-3 activation-associated apoptotic cell death in differentiated RGC-5 cells. Although it is not clear whether OPA1 release contributes to signaling events leading to caspase-3 activation in RGC degeneration or not, it is possible that OPA1 release is critical in apoptotic RGC death induced following elevated hydrostatic pressure because growing evidence indicates that OPA1 regulates mitochondrial morphology and apoptosis in many cell types [15,17,25,60,63,68-71].

Mitochondria-mediated apoptotic cell death has been reported in RGC-5 cells following serum deprivation, elevated hydrostatic pressure or oxidative stress [27,65,72,73]. It is not clear whether mitochondrial fission and OPA1 release that are seen following elevated hydrostatic pressure are associated with the mechanism of cell death in RGC-5 cells following other stresses. However, we recently found that serum deprivation significantly triggers mitochondrial fission at 12 h (unpublished data) before inducing apoptosis in RGC-5 cells as shown by Charles el al. [66]. Hence, it is possible that mitochondrial fission or OPA1 release induced by elevated hydrostatic pressure may be an early or a parallel event of mitochondria-mediated cell death in RGC responses.

Recently, it has been demonstrated that increased OPA1 reduces cytochrome C release, mitochondrial dysfunction, and cell death induced by intrinsic stimuli without interfering with activation of the mitochondrial gatekeepers, the multidomain proapoptotics BAX and BAK [74]. Moreover, a recent study has been demonstrated that increased OPA1 protects from apoptosis by preventing cytochrome C release and mitochondrial dysfunction and that OPA1 controls cytochrome C mobilization from mitochondrial cristae and apoptotic remodeling of mitochondrial cristae [25], and cell death [25,59,60]. Thus, the present observations that pressure-induced mitochondrial fission and OPA1 release occur before the onset of apoptosis raise the possibility that preventing release may protect against RGC damage or loss in glaucoma.

## Supplementary Material

movie 1

movie 2
